# The Effect of Adjuvant Intracameral Triamcinolone Acetonide on the Surgical Results of Trabeculectomy with Mitomycin C

**DOI:** 10.4274/tjo.88785

**Published:** 2016-08-15

**Authors:** Neşe Alagöz, Cengiz Alagöz, Yusuf Yıldırım, Ceren Yeşilkaya, Çiğdem Altan, Ercüment Bozkurt, Banu Şatana, Berna Başarır, Muhittin Taşkapılı

**Affiliations:** 1 Beyoğlu Eye Training and Research Hospital, İstanbul, Turkey

**Keywords:** Intracameral triamcinolone acetonide, surgical success, trabeculectomy

## Abstract

**Objectives:**

To evaluate the effect of adjuvant intracameral triamcinolone acetonide (TA) on the surgical results of trabeculectomy with mitomycin C.

**Materials and Methods:**

All consecutive trabeculectomy cases performed in the glaucoma clinic between July 2012 and December 2013 were retrospectively reviewed from the patient charts. Only those with follow-up of 12 months or longer were included. Patients with intraoperative intracameral TA (study group, n=19) were compared to those without TA (control group, n=21) in terms of surgical success, intraocular pressure (IOP) change, medication use and complications.

**Results:**

Forty eyes of 31 patients (21 male/10 female, mean age 64.2±13.8 years) were included in the study. The mean follow-up period was 20.9±5.1 months and 20.7±6.7 months in the study and control groups, respectively (p=0.830). Baseline IOP was 26.4±9.9 and 25.2±7.6 mmHg (p=0.979), and final IOP was 12.7±2.6 and 13.6±3 mmHg in both groups respectively (p=0.226). At the final follow-up, complete success was observed in 68.4% and 52.4% of the study and control groups (p=0.349) and anti-glaucoma medication was used by 31.6% (mean number of medications: 0.79±1.2) and 47.6% (mean number of medications: 1.33±1.7), respectively (p>0.05). Bleb encapsulation, leakage, suture-lysis and hypotony rates were similar in both groups (for all, p>0.05). Cataract progression was noted in six (35.3%) and in five (26.3%) of the phakic eyes in the study and control groups, respectively (p=0.720).

**Conclusion:**

When used intracamerally, TA did not increase the complication rate in trabeculectomy surgery. Although the group that received TA showed lower IOP levels, use of fewer medications and fewer eyes requiring medication, the differences did not reach significance.

## INTRODUCTION

Trabeculectomy lowers intraocular pressure (IOP) via the creation of a fistula through which the aqueous humour can drain from the anterior chamber to the sub-Tenon’s space.^1^ Unlike other surgical procedures, the goal in trabeculectomy is for the wound to only partially heal postoperatively. In fact, complete healing of the incision is considered a failed surgery. Bleb failure can occur after trabeculectomy for various reasons, but the most common cause is fibrotic changes in the conjunctiva and episclera.^2,3,4^ Therefore, modulating wound healing has particular importance in trabeculectomy.

There are four basic stages of wound healing: 1) coagulative phase, 2) inflammatory phase, 3) proliferative phase, and 4) post-proliferative remodeling phase.^5^ Mitomycin C (MMC) and 5-florouracil (5-FU), which are currently the anti-mitotic agents most commonly used in trabeculectomy, affect the proliferative phase by reducing fibroblast proliferation and activity, thereby increasing surgical success.^5,6,7^ However, because these drugs have been associated with serious, sight-threatening complications such as corneal endothelial failure, bleb leakage, blebitis and endophthalmitis,^8,9,10^ the search continues for safer alternative methods to increase the success of trabeculectomy.

As with all ocular surgeries, medications containing corticosteroids improve the success rates of trabeculectomy.^11^ Steroid-containing drugs not only affect the inflammatory phase of wound healing (by reducing leukocyte density, distribution and function), but also affect the coagulative phase by reducing vascular permeability, and the fibrotic phase by inhibiting the release of inflammatory mediators and growth factors.^5^ Triamcinolone acetonide (TA) is a synthetic steroid in the form of white crystals in an aqueous suspension. TA has been detected in the aqueous humour at minimal concentrations for 13 months after subconjunctival application and 18 months after intravitreal application.^12,13^ Intracameral administration of TA during trabeculectomy results in high TA concentrations in both the anterior chamber and the subconjunctival space, so the anti-inflammatory and anti-fibrotic effects of TA would be expected to persist in the long term.

In this study we aimed to evaluate whether the intracameral administration of TA as an adjuvant to trabeculectomy with MMC had an impact on surgical success and to assess the possible complications associated with this method.

## MATERIALS AND METHODS

Adjuvant intracameral TA was administered routinely to patients undergoing trabeculectomy with MMC in the glaucoma clinic of our hospital between April and December 2013. Therefore, the study group included consecutive patients operated during this period who met the study criteria (TA+ group), while the control group (TA- group) comprised consecutive patients from the previous period (July 2012 to March 2013). In accordance with the Declaration of Helsinki, all patients were informed about the surgical procedures and postoperative period, and written informed consent forms were obtained from all participants.

Patients followed for at least 12 months were included in the study. Patients with neovascular glaucoma and glaucoma secondary to uveitis were excluded.

The following data were recorded from patients’ charts: age, gender, ocular and systemic diseases, history of cataract surgery or other operations; preoperative corrected visual acuity (CVA), IOP values, drugs used, lens status, findings on slit-lamp, fundus and gonioscopy examinations; and postoperative IOP values, drugs used, and complications.

Patients were evaluated before the surgery; at 1 day, 1 week, and 1, 3, 6 and 12 months after surgery; and once a year thereafter. Visual acuity was measured using Snellen chart and converted to logMAR for statistical analysis. Goldmann applanation tonometer was used for all IOP measurements. Fundus examination was done using a +90 diopter (D) lens.

At the final examination, an unmedicated IOP less than 18 mmHg was considered ‘complete success’; IOP less than 18 mmHg with any anti-glaucomatous drug was ‘partial success’; and IOP of 18 mmHg or higher was ‘failure’. IOP under 5 mmHg was considered hypotony.

### Surgical Technique

**Figure 1:**
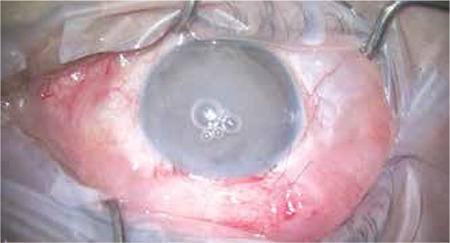
Triamcinolone acetonide is visible in the anterior chamber after postoperative intracameral injection and can be seen flowing into the subconjunctival space via the fistula

Following stabilization of the globe with a 8/0 vicryl traction suture placed in the superior limbus, a conjunctival flap was created based at the fornix. MMC was applied to the scleral surface at a concentration of 0.2 mg/mL for 2 min, then washed with at least 50 mL of saline solution. Hemostasis was achieved by cauterization when necessary. A half-thickness, 3x4 mm rectangular scleral flap was created. Paracentesis was performed through a side port opened with a 15° blade and 0.10% carbachol (Miostat, Alcon Laboratories, USA) was administered in the anterior chamber. Peripheral iridectomy was performed after removing a 1x2 mm corneoscleral block. The scleral flap was closed at 2 corners with 10/0 nylon sutures. After checking the aqueous drainage, additional sutures were placed when necessary. The conjunctiva was closed with 10/0 nylon sutures in the limbus and checked for leakage. In patients receiving intracameral TA, the procedure was concluded with the administration of 0.1-0.3 mL of 4 mg/mL TA (Kenakort-A, Deva Holding A.Ş., Çerkezköy/Tekirdağ, Turkey) into the anterior chamber through the side port (Figure 1).

All patients were treated postoperatively with topical antibiotic (0.5% moxifloxacin) 5 times a day for 2 weeks and topical 1% prednisolone acetate starting at 6 times a day for the first 2 weeks and decreasing each week for a total of 6 weeks.

### Statistical Analysis

SPSS version 20.0 software was used in all statistical analyses. Numerical variables are expressed as mean ± standard deviation (SD). Categorical variables are expressed as frequency and percentage (%). The Wilcoxon test was used for dependent intergroup comparisons of numerical variables; the Mann Whitney U test was used for independent comparisons of the two groups. Fisher’s Exact test was used for categorical variables. Results with P values less than 0.05 were accepted as statistically significant. Patients with intracameral TA (study group) and without TA (control group) were compared in terms of surgical success and complications.

## RESULTS

### Preoperative Findings

Forty eyes of 31 patients (21 male, 10 female; mean age 64.2±13.8 years) who met the study criteria were included in the study. The demographic data of the study group (n=19) and control group (n=21) are summarized in Table 1.

There was no significant difference in preoperative characteristics between the two groups (Table 1).

### Surgical Success and Change in Intraocular Pressure

**Figure 2:**
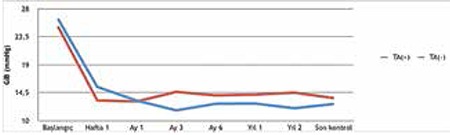
Initial intraocular pressure and postoperative changes

The mean postoperative follow-up time was 20.9±5.1 months in the study group and 20.7±6.7 months in the control group (p=0.830). At the final follow-up, complete success was observed in 13 eyes (68.4%) of the study group and partial success in 6 eyes (31.6%). In the control group, complete success was achieved in 11 eyes (52.4%) and partial success in 10 eyes (47.6%) (p=0.349). The IOP values and number of medications used preoperatively and at each postoperative follow-up in both groups are shown in Table 2. Figure 2 presents the pre- to postoperative IOP changes in the patient groups in graphic form. After postoperative month 3, the study group showed lower mean IOP and number of medications, and the number of medicated eyes was lower at the final follow-up (31.6% in the study group vs 47.6% in the control group), but these differences did not reach statistical significance.

### Complications

Table 3 summarizes the complications that occurred in each group. In the first month, argon laser suturolysis was performed in 7 eyes (36.8%) in the study group and in 6 eyes (26.6%) in the control group. In the early postoperative period, resuturation was performed in 1 eye (5.2%) of the study group due to conjunctival leakage; in the control group, conjunctival leakage was detected in 3 eyes (14.3%), 2 of which were resutured and 1 was treated with occlusion therapy. One patient in the study group was treated with systemic steroids due to hypotony and choroidal detachment in the early period. Furthermore, transient hypotony which spontaneously resolved was observed in 1 patient from both the study and control groups. Hypotony or conjunctival leakage did not occur in any of the patients in the late postoperative period. During the follow-up period, bleb encapsulation requiring needling developed in 3 eyes (15.8%) in the study group, each of which was managed with a single needling with MMC. In the control group, 4 eyes (19.1%) had encapsulated blebs; 2 of these eyes were treated once by needling with MMC, 1 eye was treated twice and 1 eye 3 times. Lens opacification occured in 6 phakic eyes (35.3%) in the study group and 5 phakic eyes (26.3%) in the control group during the follow-up period. Cataract surgery was performed in 4 eyes in both the study and control groups during the follow-up period after trabeculectomy.

## DISCUSSION

In this case series, patients with intracameral TA (study group) and without TA (control group) were compared in terms of surgical success and complications. The application of intracameral TA at the end of surgery did not result in any significant differences in the success or complication rates in our study. At the final examination after an average follow-up period of 21 months, the complete surgical success rate was 68.4% in patients treated with intracameral TA and 52.4% in the control group; partial success rates were 31.6% and 47.6% in the study and control groups, respectively. The IOP of all patients in the study group was 18 mmHg or lower at final follow-up.

Previous studies have demonstrated that minimal concentrations of TA remain in the aqueous humour for 13 months after subconjunctival application and 18 months after intravitreal application.^12,13^ Because it suppresses inflammation and reduces vascular permeability, TA is commonly used in ocular disease and surgery, usually in the form of an intravitreal or sub-Tenon injection. TA applied intracamerally during pediatric cataract surgery has been reported to provide superior control of anterior segment inflammation and fewer inflammation-related complications.^14^ It has also been shown that the use of intracameral TA does not change the IOP profile or lead to major complications in the postoperative period.^14,15^

The use of TA during glaucoma surgery was first reported in 1986 by Giangiacomo et al.^16^ They reported achieving surgical success with 4 mg of TA delivered by subconjunctival injection 0-7 days before surgery in 14 of 15 eyes determined as high-risk for episcleral scarring. In 2006, Tham et al.^17^ injected 0.03 mL of 40 mg/mL TA directly into the blebs of 6 eyes undergoing trabeculectomy. They reported promising results from this pilot study and did not observe any signs of endothelial loss or cataract progression. The results of other studies on the use of TA in trabeculectomy are summarized in Table 4. Some of the studies were unable to demonstrate any benefit related to TA,^18,19^ while positive results were reported in others.^16,17,20,21^

Although no significant difference was observed between the two patient groups in our study in terms of surgical outcomes, as of postoperative month 3 the study group showed lower mean IOP and number of medications (Figure 2, Table 2), and the number of medicated eyes was lower at the final follow-up (31.6% in the study group vs 47.6% in the control group). Although not reflected statistically, TA showed a slight beneficial effect when applied in trabeculectomy with MMC.

We observed no effect of TA delivered via intracameral route on trabeculectomy-related complications. The study and control groups showed comparable rates of encapsulation, leakage, suturolysis and hypotony (Table 3). In a case series of trabeculectomy with adjuvant intracameral bevacizumab, it was reported that despite higher rates of surgical success, bevacizumab was associated with increased conjunctival leakage in the early postoperative period.^22^ However, there are no reports of increased early or late postoperative complications of trabeculectomy associated with TA.

Steroids are known to increase cataract formation and cause substantial increases in IOP.^23^ On the other hand, trabeculectomy itself is also known to accelerate cataract development. In our case series, TA was not associated with higher risk of cataract progression in phakic eyes over the average follow-up period of 21 months. Cataract progression was observed in 35.3% of phakic eyes treated with intracameral TA and 26.3% of the control eyes, and in each group 4 eyes required cataract surgery.

The anti-neoplastic agent MMC reduces scar formation and increases surgical success after trabeculectomy by both suppressing fibroblast proliferation and decreasing collagen production in fibroblasts.^5,24^ Furthermore, MMC is toxic to both proliferative and nonproliferative cells, thus leading to apoptosis.^25^ Although MMC falls below the minimum effective concentration a few days after ocular application,^26^ it is believed that its biological effect continues for a much longer time.^27^ Due to these features, trabeculectomy with adjuvant MMC has been associated with higher rates of thin avascular blebs, bleb leakage, and resulting complications like blebitis or endophthalmitis.^8,9,10^ No increase in complication rates has been observed in studies using TA in trabeculectomy,^16,17,18,19,20,21^ suggesting that TA is safer than MMC. Similarly, we observed no serious complications related to intracameral TA administration in our case series.

A portion of the TA delivered to the anterior chamber at the end of trabeculectomy remains in the anterior chamber, while another portion flows into the sub-Tenon’s space around the scleral flap via the fistula (Figure 1). Therefore, high concentration of TA is achieved both in the anterior chamber and around the scleral flap in the early postoperative period. The portion of TA that enters the sub-Tenon’s space is released gradually, thus exerting the extended effect demonstrated in previous studies.^12^ Delivering TA via a cannula through the limbal side port created during the surgery is quite practical. With sub-Tenon or intrableb delivery, complications such as injection-related sterile necrosis or persistent TA deposits in the subconjunctival space have been reported.^17,28,29^

The basic limitations of this study are that it was retrospective and the patient number was small. Because the study was conducted retrospectively, the TA dose administered could not be completely standardized and ranged from 0.1 to 0.3 mL.

## CONCLUSION

Administration of intracameral TA during trabeculectomy did not increase the complication rate in our study. However, TA did not have a significant effect on long-term surgical success. Nevertheless, despite statistical nonsignificance, the group that received TA exhibited positive trends such as lower IOP levels, fewer antiglaucomatous drugs used postoperatively and fewer cases requiring medication. Further studies on this topic are needed.

### Ethics

Ethics Committee Approval: A retrospective study, Informed Consent: It was taken.

Peer-review: Externally peer-reviewed.

## Figures and Tables

**Table 1:**
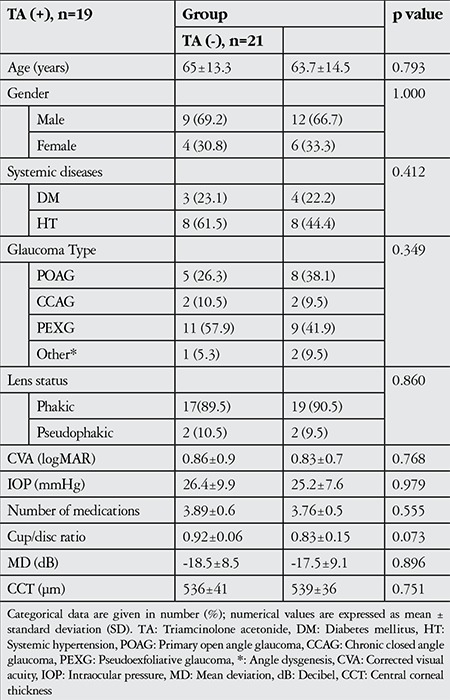
Patients’ demographic characteristics and preoperative findings

**Table 2:**
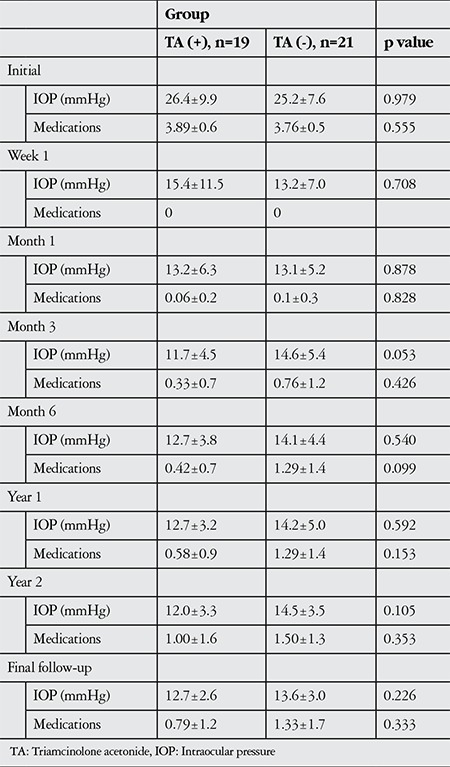
Mean intraocular pressure and number of medications in the patient groups

**Table 3:**
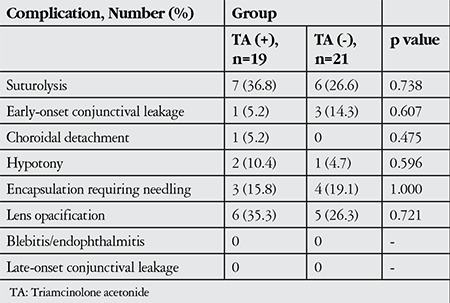
Postoperative complications observed in the two patient groups

**Table 4:**
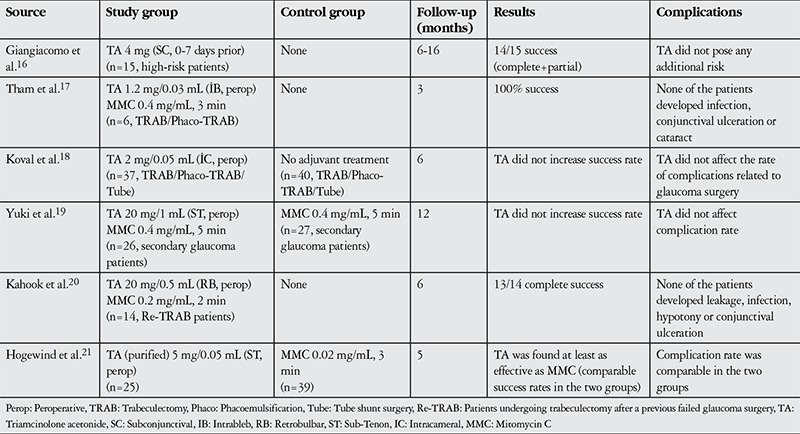
Studies using triamcinolone acetonide as an adjuvant in trabeculectomy surgery
